# News and Miscellany

**Published:** 1904-12

**Authors:** 


### News and Miscellany.

---

The American Public Health Association, composed, mind you, of sanitarians, lay and professional, will meet at Havana, Cuba, January 9, 10, 11, 12, and 13, prox.

---

Hale and Hearty at 84.—By a clerical error in the report of the Tuberculosis Congress at St. Louis, Dr. A. N. Bell, the veteran sanitarian who contributed a paper on "Stamina," was said to be prevented by "feebleness" from being present. It should have been "deafness." Dr. Bell is by no means feeble, and is likely to live to be an old man. We hope so.

---

WILL REPAIR YOUR ELECTRIC MACHINES.—Static and all electrical medical apparatus put in running order. I am also agent for electrical and X-ray apparatus. Oliver Brush, 710 Colorado Street, Austin, Texas.

---

GOLD MEDAL TO LAMBERT PHARMACAL Co., St. Louis, was awarded by the World's Fair Exposition for the excellence of their famous laboratory products, headed by the incomparable and indispensable Listerine.

---

DOCTORS CHAS. O. FARRINGTON and J. H. Evans, of Palestine, Texas, suffered a severe loss by fire on November 14th. Dr. Farrington narrowly escaped with his life, and was forced to jump from the second-story window.

---

THE STATE WON THE CASE before the Civil Court of Appeals as to the title to the ground on which the quarantine station at Galveston is built. The Federal Government claimed it. They will appeal to court of last resort.

---

DANGER IN THE BARBER SHOP.—Shave yourself with one of the famous Shumate dollar razors. Sold all over the world. Sent, post paid, as a premium for subscription to the Famous "Red Back." Razor, $1; "Red Back," $1; both for $1.50. Personal checks go.

---

SURRENDERED.—Certain mighty men of the East, split off from the American Congress on Tuberculosis in 1902, because laymen were admitted to membership, claiming that it should be composed of physicians only. The bolters had a meeting in New York recently and issued an invitation to laymen to join them. That is sensible, but why kick about it when the American Congress—the pioneers—did it?

---

NEW ORLEANS POLYCLINIC.—Eighteenth annual session opens November 7, 1904, and closes May 10, 1905. Physicians will find the Polyclinic an excellent means for posting themselves upon modern progress in all branches of medicine and surgery. The specialties are fully taught, including laboratory and cadaveric work. For further information, address New Orleans Polyclinic, postoffice box 797, New Orleans, La.

---

The Report of the Superintendent of the State Lunatic Asylum at Austin for the year ending August 31, 1904, shows: Patients on hand August 31, 1903, 1177; admitted during the year, 251; returned from furloughs, 26; making a total treated (825 males, 665 females) 1490. Died, males, 41; females, 38; total, 79. Remaining August 31, 1904, 1172. Death rate, 5.53, as against 6.08 in 1903. Cost of maintenance, per capita, $142.61.

---

The "Texas Medical Journal" has been so satisfactory to us as an advertising medium during the past year that you may continue our advertisement until further notice, at the same terms as charged us this year. We assure you it is a pleasure to be able to say this for your valued journal, and hope that both you and the Texas Medical Journal will enjoy every success during the coming year. The Antikamnia Chemical Company.

---

A well known Indiana man
One dark night last week,
Went to the cellar with a match
In search of a gas leak.
(He found it.)

John Welch by curiosity
(Dispatches state) was goaded;
He squinted in his old shotgun
To see if it was loaded.
(It was.)

A man in Macon stopped to watch
A patent cigar clipper;
He wondered if his finger was
Not quicker than the nipper.
(It wasn't.)

A Maine man read that human eyes
Of hypnotism were full;
He went to see if it would work
Upon an angry bull.
(It wouldn't.)

—*San Francisco Bulletin.*

